# Chelating agent-assisted in situ LDH growth on the surface of magnesium alloy

**DOI:** 10.1038/s41598-018-34751-7

**Published:** 2018-11-06

**Authors:** T. N. Shulha, M. Serdechnova, S. V. Lamaka, D. C. F. Wieland, K. N. Lapko, M. L. Zheludkevich

**Affiliations:** 1 MagIC-Magnesium Innovation Center, Institute of Materials Research, Helmholtz-Zentrum Geesthacht, Max-Planck-Straβe 1, 21502 Geesthacht, Germany; 2 Faculty of Chemistry, Belarusian State University, Nezavisimosti Avenue 4, 220030 Minsk, Belarus; 3 Faculty of Engineering, University of Kiel, Kaiserstrasse 2, 24143 Kiel, Germany

## Abstract

*In situ* formation of layered double hydroxides (LDH) on metallic surfaces has recently been considered a promising approach for protective conversion surface treatments for Al and Mg alloys. In the case of Mg-based substrates, the formation of LDH on the metal surface is normally performed in autoclave at high temperature (between 130 and 170 °C) and elevated pressure conditions. This hampers the industrial application of MgAl LDH to magnesium substrates. In this paper, the growth of MgAl LDH conversion coating directly on magnesium alloy AZ91 at ambient conditions (25 °C) or elevated temperatures is reported in carbonate free electrolyte for the first time. The direct LDH synthesis on Mg alloys is enabled by the presence of organic chelating agents (NTA and EDTA), which control the amount of free and/or hydroxyl bound Mg^2+^ and Al^3+^ in the solution. The application of the chelating agents help overcoming the typical technological limitations of direct LDH synthesis on Mg alloys. The selection of chelators and the optimization of the LDH treatment process are supported by the analysis of the thermodynamic chemical equilibria.

## Introduction

Magnesium alloys have attracted a lot of attention nowadays, thanks to the low density in combination with reasonable mechanical properties. This attribute makes them promising as structural materials for vehicles and their components as well as for the electronics industry. However, the corrosion resistance of magnesium alloys is not always sufficient, limiting the use of Mg-based materials by different industries, especially when they are supposed to come into contact with aggressive corrosive environments. Different protection technologies are already in use in order to increase the corrosion resistance of magnesium alloys, e.g. conversion treatment^1–3^ polymeric coatings^4,5^, plasma electrolytic oxidation (PEO) treatment^6–9^, and combinations thereof demonstrate the highest protection efficiency^10^. One central idea is based on a formation of a good barrier coating, which prevents the penetration of moisture and other corrosive agents down to the metal surface. However, even strong passive protection cannot reliably serve for the full expected service life, since service-induced defects lead to the damage of the barrier. This can result in a strong localized corrosion, starting at the defects and propagating under the coating at the layer-metal interface. Therefore, an additional active protection and a stable interface are very important. However, the most effective and well-known chromate-based treatments are strictly banned on account of toxicity for humans and danger to the environment.

Recently, layered double hydroxide (LDH)-based conversional treatments have shown great potential for corrosion protection of aluminium- and magnesium-based alloys^11–17^. LDH is a hydrotalcite-like compound, which is typically composed of positively-charged mixed metal *M*^II^-*M*^III^ hydroxide layers and galleries filled by the charge compensating anions (*A*^*y*−^) and water molecules^18–20^. The main advantage is that corrosion inhibitors can be intercalated into LDH galleries as charge compensating anions, which can be used for active corrosion protection *on demand* in the presence of corrosion-relevant triggers. Hence, LDH-based conversion layers can be effective in delaying the corrosion reaction by entrapping the corrosive anions, such as chlorides, into their interlayer and working as self-healing protective coatings, releasing the corrosion inhibitors when required^21–23^.

In comparison with aluminium alloys, LDH synthesis on magnesium causes a lot of problems because of the high reactivity of the substrate leading to Mg(OH)_2_ formation. As a first approach, synthesis of LDHs on Mg alloys was performed from the solutions containing pre-formed LDH particles. For example, Wang *et al*. firstly synthesized the LDH particles, then transferred the resulting slurry to an autoclave where Mg alloy AZ31 plates were immersed, and left the synthesis at 125 °C for 12 hours. The resultant films were modified with a hydrophobic silane coupling agent^24^. The coating revealed the presence of both hydrotalcite (LDH) and hydromagnesite phases. The hydrophobization of the coated surface was found to improve the corrosion resistance of such LDH coating. Despite improved corrosion behaviour, the main disadvantage of the LDH growing schemes discussed above is low adhesion between the LDH layer and the substrate when compared to that of the LDH intrinsically grown as a result of conversion reaction. The direct synthesis of LDH on the surface overcomes this problem by using the components from both the substrate and the electrolyte.

A number of different approaches has been used in order to obtain LDH layers directly on magnesium alloys. For example, Nakamura and Kamiyama have used LDH-based coatings for corrosion protection of magnesium alloy (AZCa612 and AZ31 respectively)^25,26^. In both works, the formation of LDH film was achieved by steam-coating treatment, and improvement in corrosion behaviour was demonstrated. Chen *et al*. have investigated the MgAl LDHs growth *in situ* on bare AZ31 Mg alloy via a two-step process (precursor film based on MgAl_2_(OH)_8_·xH_2_O and Mg_2_Al(OH)_7_ formation with following recrystallization to MgAl LDH-carbonate in the presence of bubbling CO_2_) and have suggested a possible mechanism of MgAl LDH formation^27–30^ as well as an exchange mechanism in NaCl^31^. The main limitation of this approach is that LDH was prepared in the form of MgAl LDH-carbonate. The carbonate anions have a high affinity to the LDH layers and hardly exchangeable with functional species (e.g. inhibitors) that are to be used for corrosion protection. Lin *et al*.^32^ has demonstrated the possibility of forming MgAl LDH conversion layer on AZ91 alloy also in aqueous ${{\rm{HCO}}}_{3}^{-}{/\mathrm{CO}}_{3}^{2-}$ media. Moreover, the authors have shown that MgAl LDH-carbonate can be exchanged with LDH-Cl in a corrosive environment, demonstrating the effective protection of Mg alloy against corrosion. More recently, the same authors proposed a modified procedure to grow LDHs, based on the fact that during the reaction of Mg substrate with H_2_O, the alkalinity of the solution increases with an increase in the immersion time. Therefore, instead of growing LDHs over a long period of time, they have proposed a two-step methodology with an overall reduced processing time, where a first step in the solution enriched with ${{\rm{CO}}}_{3}^{2-}{/\mathrm{HCO}}_{3}^{2-}$ at pH 6 is followed by a second step where pH was increased to 11.5^33^. Recently, the formation of MgAl LDH-carbonate was also optimized for the treatment of magnesium alloys, covered with PEO (plasma electrolytic oxidation) layer^34–38^. The main disadvantage of the PEO treatment is that layer properties are strongly compromised by the presence of defects and cracks, formed during the discharges. MgAl LDH, formed on the PEO-treated surfaces, helped overcome this problem (sealing of pores and cracks took place).

Overall, although significant progress is achieved in LDH formation on Mg substrates, two main drawbacks remain:

1. The low adhesion between substrate and LDH layer in the case of pre-formed LDH use, and
2. LDH formation occurs under autoclave conditions, which significantly reduces the possibility of industrial applications of the suggested treatment.

The first problem can be solved via the direct synthesis of LDH on the surface, which is achieved in this work. The second drawback originates from the low solubility of magnesium compounds in the pH range, suitable for LDH formation (ca. pH 9–10 for ambient conditions)^18^. In this work, we extended the pH range of solubility of magnesium compounds using chelating agents and exploited their ability to promote controlled dissolution of AZ91 magnesium alloy via the formation of soluble chelating complexes. Moreover, carbonate free electrolyte was used in order to avoid LDH contamination with these hardly exchangeable anions, limiting LDH application for industrial needs^39^. Recently, an experimental database listing more than 150 chemical compounds that inhibit or promote the dissolution of pure magnesium and its alloys has been set^40^. Three chelators (salicylic acid, NTA, and EDTA) were chosen to prove an idea of LDH growth in relatively mild conditions (ambient pressure). As a result, the formation of MgAl LDH was found to be possible at mild temperature and ambient pressure conditions suitable for further industrial upscaling.

## Materials

Aluminium nitrate nonahydrate (Al(NO_3_)_3_∙9H_2_O, ≥98%, CarlRoth GmbH, Germany), sodium nitrate (NaNO_3_, ≥99%, Merk KGaA, Germany), sodium hydroxide (NaOH, ≥99%, Merk KGaA, Germany), salicylic acid (SA, C_7_H_6_O_3_, ≥99%, Sigma-Aldrich Chemie GmbH, Germany), ethylenediaminetetraacetic acid (EDTA, C_10_H_16_N_2_O_8_, min 99%, AppliChem GmbH, Germany), and disodium salt of nitrilotriacetic acid (NTA, C_6_H_7_NNa_2_O_6_, ≥99%, Sigma-Aldrich Chemie GmbH, Germany).

## Methods

LDH was grown on magnesium alloy AZ91 with the following elemental composition [wt. %]: 8.60 Al, 0.64 Zn, 0.22 Mn, 0.027 Nd, 0.0073 Si, 0.0053 Sn, 0.0046 La, 0.0023 Cu, 0.0010 Fe, 0.0003 Ni, 0.00076 Be, 0.0007 Ti, <0.02 Th, <0.0009 Ce, <0.0006 Zr, <0.0004 Pb, <0.0002 Pr, <0.0001 Ag, Mg balance. The size of coupons was equal to 15 × 15 × 4 mm. Prior to LDH treatment, the surface of each sample was ground with SiC paper up to 1,200 grit, rinsed with deionized water, and dried by a stream of cold air.

The chelating agents (salicylic acid, EDTA, and NTA) were separately dissolved in deionized water (different concentrations of chelating agents were prepared, as will be indicated later in the description of each experiment) and pH of the solution was adjusted to 7.0–7.4 using 0.1 M and 1 M NaOH solution, where necessary. Al(NO_3_)_3_ (0.05 M) and NaNO_3_ (0.25 M) were added to individual chelating agent solutions for the LDH formation (following the standard ratio of inorganic compounds previously adopted for LDH synthesis on aluminium alloys)^41–45^. The pH of the resultant solutions was adjusted to 8.0 ± 0.1, 9.0 ± 0.1, 10.0 ± 0.1, and 11.0 ± 0.1 using NaOH (0.1 M and 1 M).

AZ91 samples were immersed in the treatment bathes with chelating agents at temperature from 25 ± 5 °C to 95 ± 5 °C under continuous stirring. The synthesis was performed during either 15 minutes, or 1, 3, 6 or 48 hours, as indicated in the respective experimental results below.

## Techniques

### Grazing incidence X-ray diffraction (GID)

The coating were examined on a diffractometer from Seifert using an X-ray energy of 8kev (Cu Kα radiation) provided by a copper anode. The measurements were carried out in a 2 theta range from 8° to 24° in steps of 0.01°. The incident angle was set, fixed at 0.2°. The angular range was measured in two steps, employing a one-dimensional detector with an acquisition time of 100 s for each point.

### X-ray diffraction (XRD)

The crystallographic analysis was performed using Bruker D8 Advance diffractometer. The measurements were carried out with Cu Kα radiation in the range of 2 theta from 5 to 30° (exposure time 1 s, step 0.02°) and from 59 to 65° (exposure time 10 seconds, step 0.02°).

### Scanning electron microscope (SEM)

The morphological characterization was performed using TESCAN VEGA3 scanning electron microscope. The following conditions were used for image acquisition: HV 8.0 kV, BI 6.00.

*Hydra-Medusa* software^46^ was used to estimate the possibility of LDH formation on the surface of magnesium alloy AZ91 with and without three chelating agents.

## Results and Discussion

**Figure 1:**
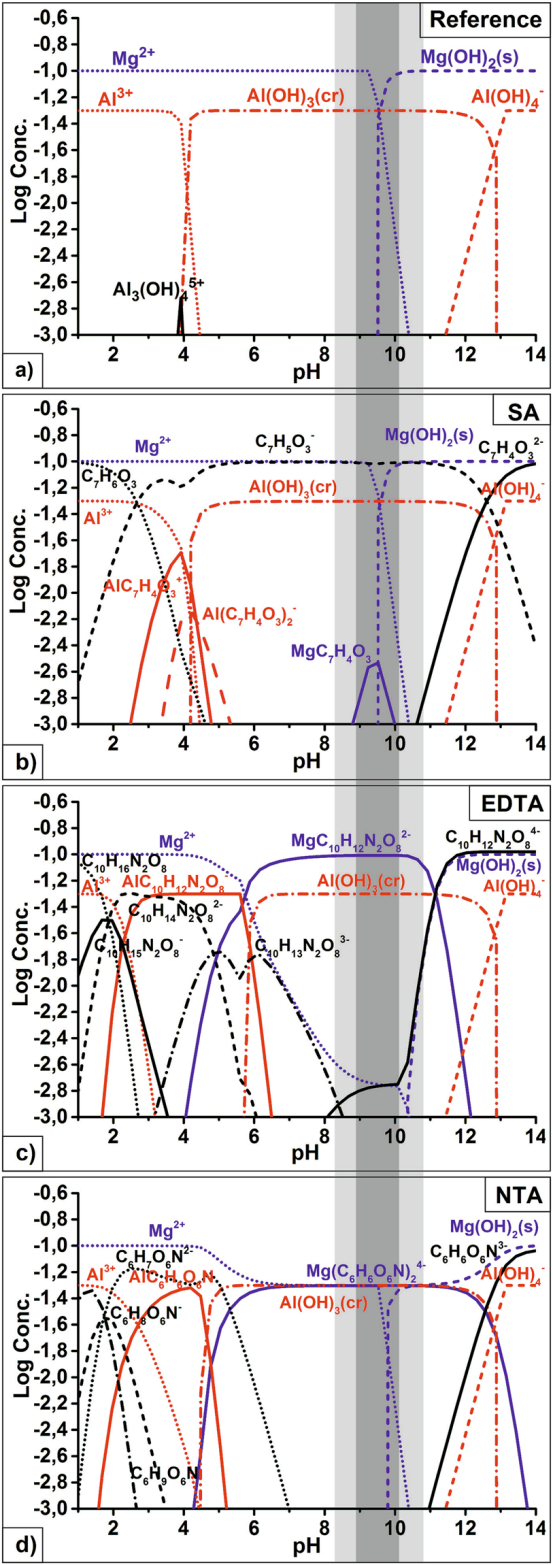
Thermodynamic calculation of the equilibrium composition of relevant species in the solutions containing different complexing agents. The pH region highlighted in grey is the most suitable for LDH formation in the form of powder following the literature data.

The investigation and optimization of LDH formation on the surface of AZ91 was performed in the solutions containing either salicylic acid, EDTA, or NTA. The results were compared with reference synthesis without organic additives. In order to estimate the possibility of LDH formation on the surface of AZ91 in the presence of chelating agents (the availability of soluble Mg containing compounds), the simulation of possible chemical equilibria was performed using the Hydra-Medusa software^46^. The following concentrations were used for calculations: chelating agents 0.1 М, Al(NO_3_)_3_ 0.05 М, NaNO_3_ 0.25 М; the estimated concentration of Mg^2+^ formed in the diffusion layer due to the dissolution of Mg substrate was postulated as 0.1 М based on previous measurements of local concentration of Mg^2+^ with ion-selective micro-electrodes^47^. The results of the Hydra-Medusa simulation are shown in Fig. 1(a–d).

The stability constants of complexes formed between salicylic acid, EDTA, and NTA chelating agents and metal cations, Mg^2+^ and Al^3+^, were updated or added into the Hydra-Medusa software based on the data provided by the literature^48–52^. The used values of logK_Mg_ and logK_Al_ along with solubility products are given in Table 1. Regrettably, neither up-to-date stability constants nor up-to-date solubility product of MgAl LDH are available; hence, these were not taken into account during the performed simulation. However, from previous works, it is known that the pH range, mostly suitable for MgAl LDH formation in powder form, is between 9 and 10 (highlighted in Fig. 1 in dark grey colour, while light grey indicates the extended pH range of LDH formation in those conditions)^18^.

**Table 1:** Stability constants of selected ligands with aluminium and magnesium ions.

Ligand^48–52^	logK_Al_	logK_Mg_
OH^−^	ML/M∙L 9.01	ML/M∙L 2.58
	ML_2_/M∙L^2^ 18.7	
	ML_3_/M∙L^3^ 27.0	pKsp Mg(OH)_2_ = 11.25
	ML_4_/M∙L^4^ 33.0	
	pKsp Al(OH)_3_ = 32.89	
Salicylate	ML/M∙L 12.9	ML/M∙L 4.7
	ML_2_/M∙L^2^ 23.2	
	ML_3_/M∙L^3^ 29.8	
EDTA	ML/M∙L 16.11	ML/M∙L 8.64
NTA	ML/M∙L 11.4	ML/M∙L 5.50
		ML_2_/M∙L^2^ 10.2

Based on the simulation results in Fig. 1a, the formation of aluminium hydroxide Al(OH)_3_ takes place at pH >4 if no chelating agent is added. In this case, since the values of the stability constants and the solubility product for aluminium hydroxide considerably exceed that for magnesium hydroxide, Mg^2+^ ions stay in the solution in an unbound state. When pH increases to above 9.5, an excess of hydroxide ions develops in the solution, leading to the formation of Mg(OH)_2_. Up to pH of about 13, Al^3+^ is present in the system as insoluble Al(OH)_3_. Above this value, an excess of OH^−^ is present, and aluminium gets dissolved as ${\mathrm{Al}(\mathrm{OH})}_{4}^{-}$.

When chelating agents are introduced into the system, formation of soluble aluminium-ligand complexes takes place. These complexes are typically stable to at least pH 4.5 to 6.0. The precipitation of Al(OH)_3_ occurs only at higher pH values. This fact, however, does not prevent the formation of LDH on Al surface neither with Mg^2+^ nor with Zn^2+^. Both precipitate at pH above 9, which means that Al(OH)_3_ is less stable than (Zn/Mg)Al LDH.

Figure 1b shows the thermodynamic possibility of the formation of the magnesium salicylate (MgC_7_H_4_O_3_) in the pH range of 8 to 9.6 and its abrupt transformation to Mg(OH)_2_ at pH >9.6. The addition of salicylate delays the formation of Mg(OH)_2_ by only 0.1 pH unit compared to reference electrolyte, from pH 9.5 to pH 9.6. The concentration of free Mg^2+^ cations in the solution at pH range of 9.6 to 10.3 — favourable for LDH precipitation — is low at these conditions. Therefore, LDH precipitation is not likely to occur in the presence of salicylate ions.

Magnesium complex with EDTA (MgC_10_H_12_N_2_O_8_^2−^) exists in a wider pH range from 4 to 12 (Fig. 1c). The concentration of the magnesium-EDTA complex increases up to the pH value of 7, and can be associated with the competition between EDTA and hydroxyl. At a pH from 7 to 10.5, the concentration of the complex formed by EDTA and magnesium reaches its maximum. Above these pH values, the chelating ion is exchanged by hydroxyl forming Mg(OH)_2_. The addition of EDTA delays the formation of Mg(OH)_2_ by 0.8 pH units, from pH of 9.5 in the reference electrolyte to pH of 10.3 in the presence of EDTA. Importantly, ca. 2% Mg^2+^ is bound neither in Mg-EDTA nor in Mg(OH)_2_ complex at pH of 9.6 to 10.3, the range favourable for LDH precipitation. This amount of Mg^2+^ formed due to Mg substrate dissolution is also available for LDH formation, while in the reference and salicylate cases, most of the Mg^2+^ is bound in Mg(OH)_2_ in this pH range (Fig. 1a). The Mg^2+^ and Mg-EDTA complex co-existing at pH of 9.6 to 10.3 can favour precipitation of LDH.

Disodium salt of nitrilotriacetic acid forms complexes with magnesium $({\rm{Mg}}{({{\rm{C}}}_{{\rm{6}}}{{\rm{H}}}_{{\rm{6}}}{{\rm{O}}}_{{\rm{6}}}{\rm{N}})}_{2}^{4-})$ at pH from 4.5 to 13.8, as shown in Fig. 1d. However, at pH above 10, the concentration of ${\text{Mg}}^{2+}$ decreases, while the formation of magnesium hydroxide starts at pH higher than 10.0. Again, it is important that a considerable amount of unbound Mg^2+^ is present in the solution at pH 1 to 10.4. Even when Mg-NTA complex is formed, unbound Mg^2+^ constitutes up to 50% of all magnesium species. Once Mg(OH)_2_ starts forming, 50% of Mg species are still constituted by Mg-NTA complex (Mg(C_6_H_6_O_6_N)_2_^4−^) up to pH above 12 when Mg(OH)_2_ fully binds all magnesium species. The presence of free Mg^2+^ and magnesium reversibly bound in $({\rm{Mg}}{({C}_{{\rm{6}}}{{\rm{H}}}_{{\rm{6}}}{{\rm{O}}}_{{\rm{6}}}N)}_{2}^{4-})$ complex in pH range from 9.6 to 10.3 can promote the formation of LDH.

From the overall analysis of Fig. 1, one can conclude that unlike in the reference and salicylate cases, in EDTA- and NTA-containing solutions, LDH is likely to precipitate because of the availability of free Mg^2+^ and Mg-Lig complexes at pH above 9.6.

Although informative and visual, thermodynamic simulations presented in Fig. 1 possess only limited predictive power because the equilibrium constants used are only valid at room temperature. Further uncertainties arise as no stability constants and solubility products of MgAl LDH are available. Besides, the thermodynamic diagrams presented are not able to provide any information about the precipitation/complexation kinetics. Nevertheless, the results of the simulations shown in Fig. 1 demonstrate the existence of unbound Mg^2+^ and reversibly bound in complex magnesium at pH above 9.6. The diagrams also explain the general principle how the chelating agents influence chemical equilibria and compete with OH^−^ ions for binding Mg^2+^ species. The chelating agents considered delay the formation of Mg(OH)_2_ (in pH scale) and are expected to facilitate the precipitation of MgAl LDH.

**Figure 2:**
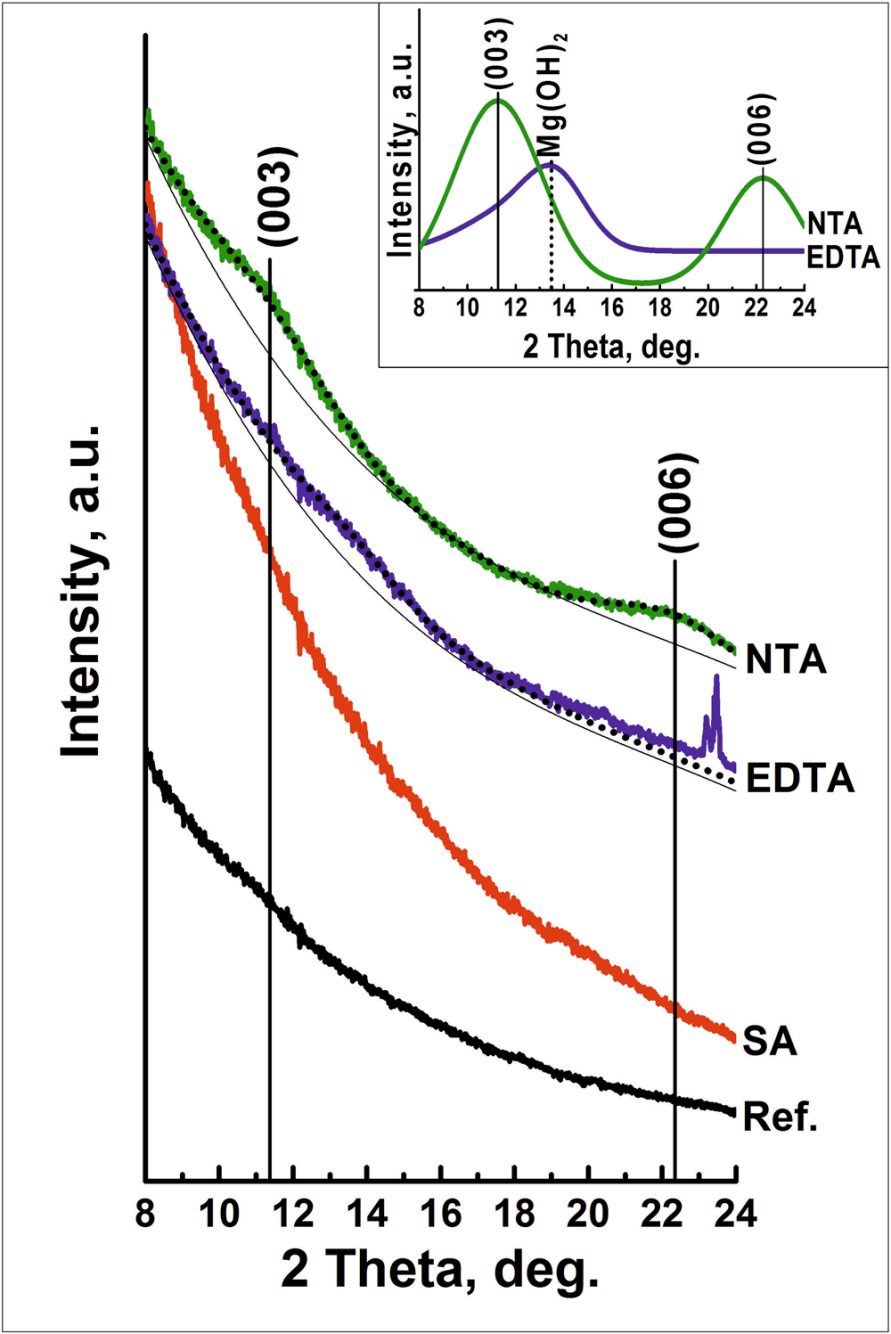
Grazing incidence XRD pattern of AZ91 surface after 48 hours in reference, salicylic acid, EDTA, and NTA sodium salt disolutions at room temperature. The data are shifted vertically for clarity. The inset shows the Gaussian polynomial fit of NTA and EDTA patterns.

In order to verify the thermodynamic calculations for the direct LDH formation on the surface of AZ91, experimental LDH growth in the presence of chelating agents was performed at room temperature (25 °C) during 48 hours. Corresponding grazing incidence X-ray diffraction patterns are shown in Fig. 2.

**Figure 3:**
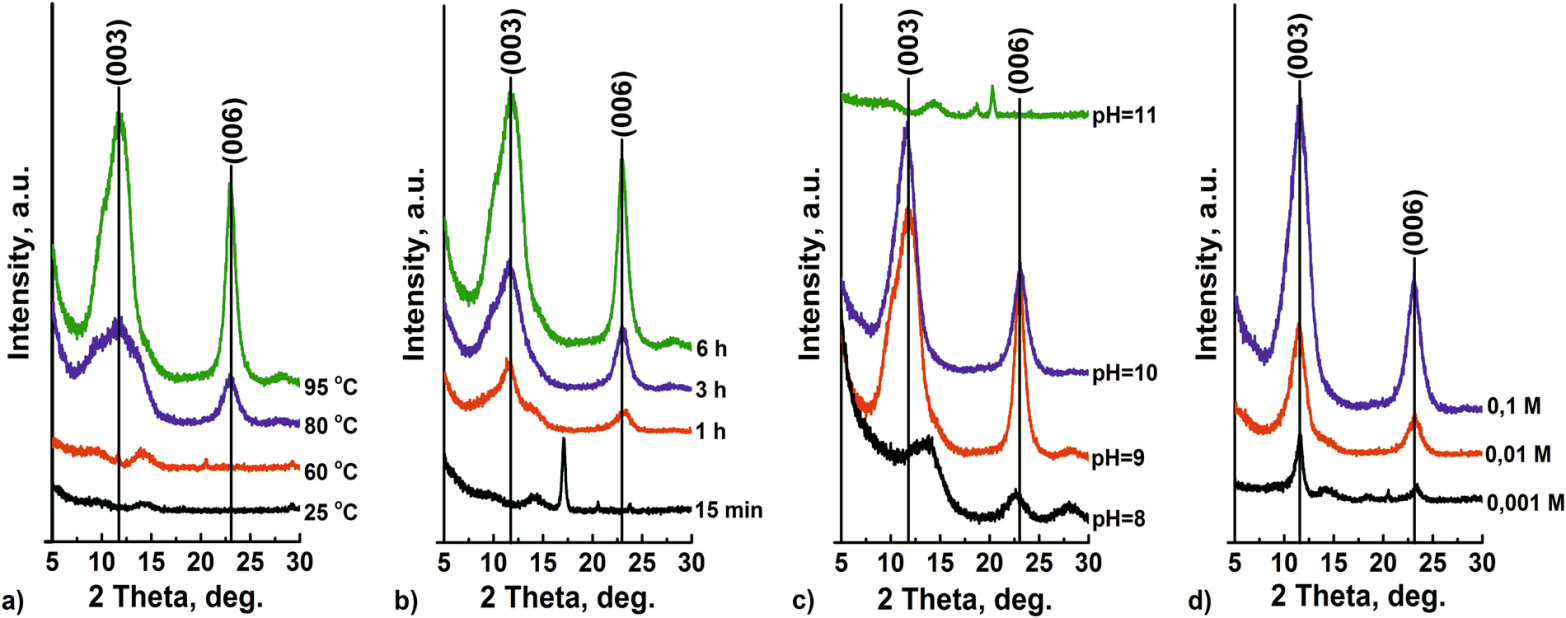
XRD pattern of AZ91 magnesium alloy after the treatment in NTA solution as function of: (**a**) temperature *(initial NTA concentration 0.1* *M, pH* = *9, 6* *h)*, (**b**) time *(0.1* *M, pH* = *9, t* = 95 *°C)*, (**c**) pH (*0.1* *M, t* = 95 *°C, 6* *h*), and (**d**) concentration *(pH* = *10, t* = 95 *°C, 6* *h)*. The data are shifted vertically for clarity.

In order to access the peak positions, a combination of two Gaussian and a polynomial function —accounting for the background — was fitted to the data. The parameters of the polynomial of third order were determined on the bases of an AZ91 reference sample without surface treatment. The result of the fitting is presented in the Inset in Fig. 2. It can be seen that the data show very broad and weak peaks. The 2 theta positions determined by the fitting are 11.25° and 22.26° for the NTA treatment. These peaks can be attributed to LDHs loaded with hydroxide/carbonate in interlayers. In the frame of this work, we assign this peak to MgAl LDH-hydroxide, since no additional carbonates were added to the solution. For the sample treated in EDTA-containing solution, two broad peaks at 11.56° and at 13.66° are also observed. The lower peak can be attributed to LDH loaded with hydroxide/carbonates (similar to the sample obtained in NTA solution, we consider this peak as LDH-hydroxide) whereas the second peak is associated with magnesium hydroxide formation. (006) LDH peak for the sample treated in EDTA solution is not visible from the patterns presented. Overall, these results show that LDH growth on the surface of AZ91 at 25°С is possible; however, the kinetics of this process are very slow. The thickness of LDH flakes, calculated using the Scherrer equation, can respectively be estimated as 4.5 and 4.2 nm for NTA and EDTA. In order to accelerate the LDH growth, the treatment in NTA containing electrolyte was performed at elevated temperatures. The synthesis was performed at 25, 60, 80, 95 °C. It can be clearly seen that with an increase in temperature, the intensity of the diffraction peaks increases, suggesting an extended LDH formation (Fig. 3a). The growth kinetics of LDH are significantly accelerated at temperatures higher than 60 °C.

The detailed investigation of the effects of chelating agent concentrations, pH of the solutions, and the duration of synthesis was also performed for the solutions of NTA disodium salt, and is presented in Fig. 3. Syntheses were carried out using NTA solutions with concentrations equalling 0.001, 0.01, and 0.1 M (Fig. 3d). It can be seen that the highest Bragg reflections are observed for the highest concentration of the chelating agent in the solution. It is also demonstrated that the optimum pH for LDH synthesis is between 9 and 10, while at pH values of 8 and 11, the formation of LDH was not observed (Fig. 3c). This result is in good agreement with previously published MgAl LDH powder synthesis^18^ and with Medusa thermodynamic predictions. Syntheses were carried out during 15 minutes and 1, 3, and 6 hours (Fig. 3b). The results show that the (003) Bragg reflection from LDH is detectable after 1 hour of treatment.

**Figure 4:**
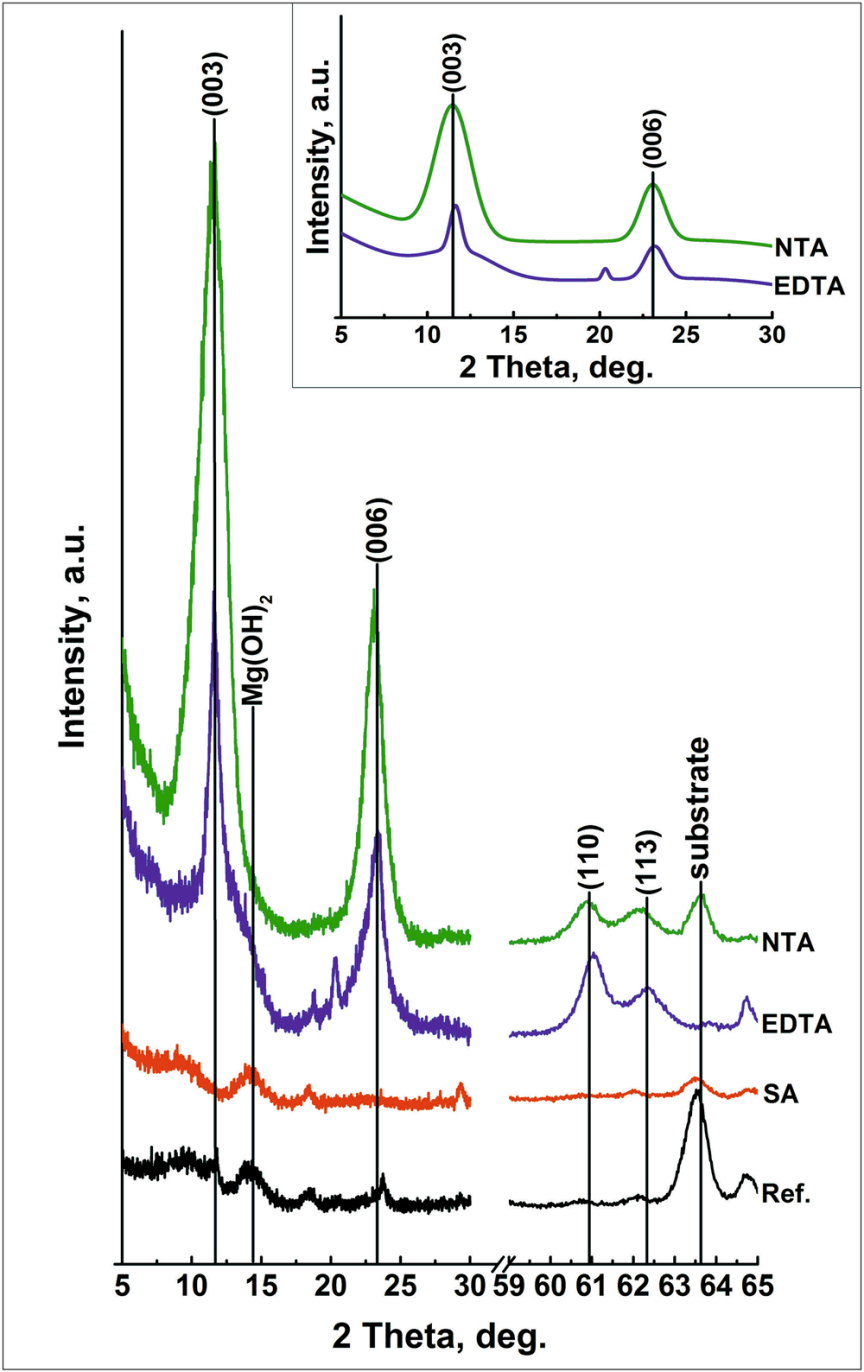
The XRD patterns of the magnesium alloy AZ91 after the treatment in reference solution of *0.05* *M Al(NO*_3_)_3_*∙9H*_2_*O* + *0.25* *M NaNO*_3_, and solution of sodium salts of salicylic acid, EDTA, NTA at pH 10.00 and T equal 95 °C during 6 h. The data are shifted vertically for clarity. The inset shows the Gaussian polynomial fit of NTA and EDTA patterns.

The optimized parameters, obtained for the LDH synthesis in the NTA-containing solution, were also applied to three other solutions, investigated in the current work (EDTA, salicylic acid, and reference). The LDH formation on the surface of AZ91 was done at pH value of 10, and at 95 °C during six hours under continuous stirring and ambient pressure. Figure 4 shows the XRD pattern of magnesium alloy AZ91 treated in reference, salicylic acid, EDTA, and NTA solutions.

The typical diffraction pattern related to LDH structures intercalated with hydroxide can be observed with peaks at 11.5° and 23.5°, as well as at 61° and 62.5°, for the samples treated in NTA and EDTA solution. These peaks can be associated with (003), (006), (110), and (113) reflections of LDH. A shoulder in the pattern of the EDTA sample, next to the (003) reflection of LDH, can be attributed to the presence of hydroxides. The thickness of LDH crystals, calculated using Scherrer’s equation, is estimated to be 10 and 20 nm for NTA and EDTA respectively.

**Figure 5:**
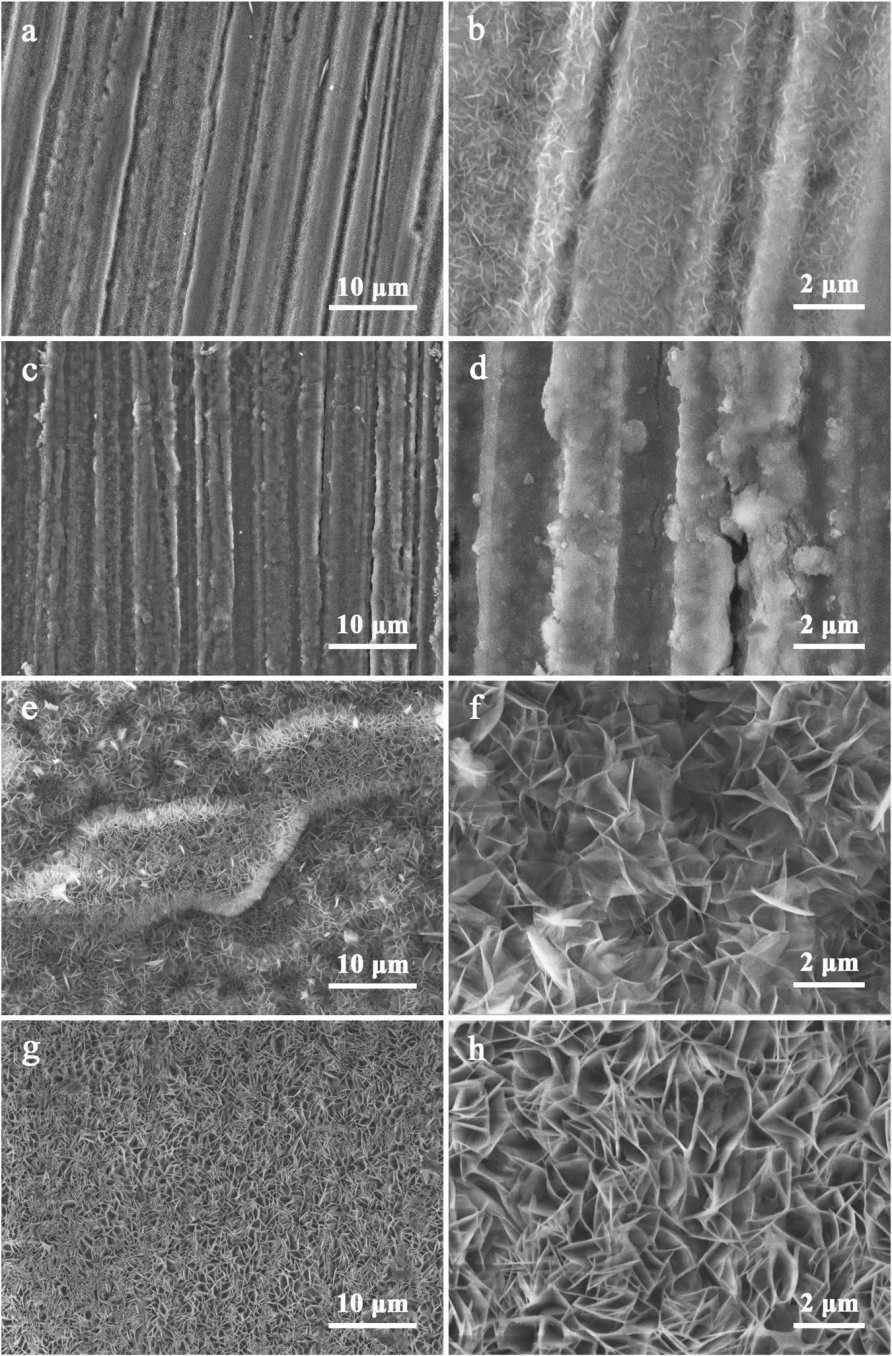
SEM micrograph of AZ91 alloy treated in a reference solution (**a,b**) and in solutions containing chelating agents: sodium salt of salicylic acid (**c,d**), EDTA (**e,f**), and NTA (**g,h**) at pH 10 and 95 °C during six hours.

The SEM results presented in Fig. 5 also confirm the formation of flake-like structures typical for LDH layer morphology on the surface of the magnesium alloy AZ91 treated with the EDTA and NTA solution. The comparison of the XRD patterns and SEM micrographs of the flakes formed on the sample obtained in a reference solution (Fig. 5a,b) shows that the structures formed correspond to hydroxide rather than LDH structure.

Overall, based on the XRD and SEM results obtained, the LDH synthesis is successfully performed in solutions containing NTA and EDTA. This result is in good agreement with a prediction, obtained via thermodynamic calculations, and can be summarized as:

1. autoclave conditions are not required for LDH growth. It makes this synthesis attractive for further optimization to match the industrial requirements.
2. no external CO_2_/CO_3_^2−^ is present in the system. This significantly increases the chances to intercalate functional species (e.g. corrosion inhibitors) within the galleries.
3. significantly higher adhesion between LDH and substrate can be expected in comparison with electrodeposited LDH coating.

All these factors can potentially decrease the production costs, making LDH a strong competitor as conversion coating with active corrosion protection and improved adhesion. As the next step of this work, we plan to study the corrosion protection properties of the LDH layers developed (including the intercalation of selected corrosion inhibitors) and verify their adhesion to Mg substrate.

## Conclusion

This work shows the principal possibility of direct LDH growth on the surface of magnesium alloy AZ91 at room temperature and ambient pressure in carbonate free electrolyte. The key components for shifting equilibrium towards LDH growth are the chelating agents such as EDTA and — especially — NTA. They prevent separate precipitation of Mg(OH)_2_ and Al(OH)_3_ and promote formation of MgAl LDH. This is achieved via the maintenance of free Mg^2+^ or magnesium reversibly bound in Mg-Lig complexes at pH exceeding typical precipitation range of Mg(OH)_2_. What is important is that no external Mg^2+^ ions are added in the electrolyte. Instead, Mg substrate gradually dissolves (assisted by chelating agents), forms complexes Mg-Lig and Al-Lig, and then subsequently forms Mg-Al LDH growing from AZ91 surface. As expected, the formation kinetics significantly increase at elevated temperature. This work shows a major route for further condition optimization of LDH formation, since it sets the way for overcoming all significant drawbacks of LDH formation on magnesium alloys.
